# Biofilm Formation by *Streptococcus mutans* is Enhanced by Indole via the Quorum Sensing Pathway

**DOI:** 10.1264/jsme2.ME19164

**Published:** 2020-04-29

**Authors:** Tomohiro Inaba, Nozomu Obana, Hiroshi Habe, Nobuhiko Nomura

**Affiliations:** 1 Environmental Management Research Institute, National Institute of Advanced Industrial Science and Technology (AIST), Onogawa 16–1, Tsukuba, Ibaraki 305–8569, Japan; 2 Transborder Medical Research Center, Faculty of Medicine, University of Tsukuba, Tsukuba, Ibaraki 305–8575, Japan; 3 Faculty of Life and Environmental Sciences, University of Tsukuba, Tsukuba, Ibaraki 305–8572, Japan

**Keywords:** Biofilm, Quorum sensing, *Streptococcus mutans*, indole

## Abstract

Interspecies interactions among oral microorganisms in the pathogenic biofilms causing dental caries have not yet been elucidated in detail. We herein demonstrated that indole and its derivatives induced biofilm formation by *Streptococcus mutans*. Indole is an intercellular signaling molecule that is produced by oral bacteria other than *S. mutans*. The amounts of biofilm and extracellular DNA were significantly increased by the addition of indole and 4-hydroxyindole (4-HI). An examination with quorum sensing mutants showed that the induction of biofilm formation by indole and 4-HI required a quorum sensing system. These results suggest that this intercellular signaling molecule plays a role in pathogenic biofilm formation.

A wide variety of microorganisms inhabit the oral cavity (Griffen *et al.*, 2012), and the dynamics of the oral microbial community influence the onset of oral infectious disease (Lamont *et al.*, 2018). Biofilms are the most important habitat for oral microorganisms, and consist of multiple species of oral bacteria in nature. Intra- and interspecies interactions in the oral microbiota influence the composition and development of oral diseases in dynamic communities; however, the underlying mechanisms remain elusive. Dental caries, which is a typical oral infectious disease, is caused by pathogenic biofilms that form on the tooth surface (Bowen *et al.*, 2018). In biofilms, cells and self-produced extracellular polymeric substances tightly packed in the community restrict the diffusion of microbial metabolites, such as lactic acid, resulting in the creation of an acidic microenvironment that causes dental decay. *Streptococcus mutans* plays a pivotal role in the formation of cariogenic biofilms by producing insoluble extracellular polysaccharides and lactic acid from carbohydrates, such as starch and sucrose (Hamada and Slade, 1980; Loesche, 1986). Due to the importance of *S. mutans* in dental caries, the mechanisms underlying biofilm formation have been investigated in detail. Moreover, the actual oral biofilm consists of a number of species of oral bacteria, and previous studies demonstrated that interspecies coaggregation constructed polymicrobial biofilms (Kolenbrander *et al.*, 2010). Based on the paradigm of interspecies communication among oral bacteria, interspecies signaling to *S. mutans* appears to facilitate cariogenic biofilm formation and microbial ecological interactions in the oral cavity (Nakanishi *et al.*, 2018). Signaling molecules are some of the most important factors in multispecies biofilm development, and, for example, autoinducer-2 has been identified as a universal interspecies signaling molecule that enhances multispecies biofilm formation by the oral commensal bacteria *Actinomyces naeslundii* and *Streptococcus oralis* (Rickard *et al.*, 2006). In the oral cavity, indole and its derivatives are candidates for interspecies signaling molecules. Indole is an aromatic heterocyclic molecule that is synthesized from a tryptophan by a number of oral bacteria and functions as an intercellular signaling molecule in microbial communities (Lee and Lee, 2010). Indole and its derivatives act as metabolic signals and play a role in biofilm formation (Hu *et al.*, 2010). The periodontal bacterium *Fusobacterium nucleatum* produces indole, which promotes its own biofilm formation (Sasaki-Imamura *et al.*, 2010). Moreover, indole has been detected in human saliva (Tonzetich *et al.*, 1967; Cooke *et al.*, 2003). These findings indicate that indole functions as a biofilm-related interspecies signaling molecule in the oral cavity.

In the present study, we investigated the influence of indole and its derivatives on biofilm formation by cariogenic *S. mutans*. *S. mutans* does not produce indoles as its own signal molecules and does not possess orthologous genes encoding receptors for indole, which have been detected in other bacteria (Lee and Lee, 2010). The materials and methods used are shown in Supplementary materials. Three strains of *S. mutans* were used in the biofilm formation assay with or without 500‍ ‍μM indole. The addition of indole increased biofilm formation by the three strains more than that dimethyl sulfoxide (DMSO), which was used as the solvent (Supplementary Fig. S1A). Thus, we used the most commonly used laboratory strain UA159 in subsequent experiments. Some indole derivatives, such as hydroxyindoles and isatin, are produced by many microbial species as metabolites of indoles through the actions of monooxygenase and dioxygenase (Lee and Lee, 2010). Moreover, oxidized indole derivatives have been shown to influence the bacterial behaviors of non-indole-producing bacteria similar to indole (Melander *et al.*, 2014). Therefore, the effects of four types of indole derivatives, namely, 2-hydroxyindole (2-HI), 4-HI, 6-HI, and isatin, on biofilm formation by *S. mutans* were examined (Fig. 1A). The results obtained showed that the addition of 4-HI and indole increased biofilm formation, whereas that of isatin, 2-HI, and 6-HI did not (Fig. 1A). Therefore, modifications to indole appeared to mostly abolish the biofilm induction effect; however, specific modifications, for example, 4-hydroxylation, maintained this activity. Approximately 0.3–50‍ ‍μM indole has been detected in human saliva (Tonzetich *et al.*, 1967; Cooke *et al.*, 2003). To verify that the concentrations of indole and 4-HI were sufficient to influence biofilm formation, concentration series of indole and 4-HI (0.5, 5, 50, and 500‍ ‍μM) were added to media, and the influence of these concentrations on biofilm formation was investigated. The results obtained showed that the biofilm-inducing effects of indole and 4-HI were detectable at the lowest concentration (0.5‍ ‍μM, Supplementary Fig. S1B). Therefore, the concentration of indole present in the human oral cavity appears to be sufficient to stimulate biofilm formation by *S. mutans*. Based on the appearance of biofilms in the presence of indole or 4-HI, the effects of 4-HI on biofilm formation appeared to differ from those of indole (Fig. 1A, upper panel). Confocal microscopic observations showed that indole and 4-HI increased biofilm thickness more than DMSO (Fig. 1B, C, and D). However, the bottom of the biofilm, *i.e.*, the surface of hydroxyapatite, in the presence of 4-HI was not observed, although the bottoms of the biofilms with DMSO and indole were clearly visualized by reflection microscopy (shown as gray colors in Fig. 1B and C). This signal decay was attributed to the impenetrability of the excitation laser at the deeper position of the biofilm. A highly dense biofilm structure caused reflection signal extinction. The area occupancy of biofilms in a cross-section, *i.e.*, the density of biofilm cells at a certain plane, was increased more by the addition of 4-HI (68.4±6.8%) than the DMSO control (56.5±1.7%) and indole (58.0±4.6%). These results suggest that the biofilm structure was denser under the 4-HI added condition. Thus, 4-HI appears to increase the density of biofilms and potentially reinforce their structure. Extracellular insoluble polysaccharides and extracellular DNA (eDNA) are the main components in biofilms formed by *S. mutans*, and promote their structural integrity and adhesion to substratum surfaces, respectively (Leme *et al.*, 2006; Bowen and Koo, 2011; Das *et al.*, 2011; Klein *et al.*, 2015). However, sucrose, which is necessary for extracellular insoluble polysaccharide production (Wood and Critchley, 1966), was not present in the medium used in the present study. Hence, we extracted and quantified the amounts of eDNA in biofilms to clarify whether indole and 4-HI increased eDNA release. We found significant increases in eDNA levels in the presence of indole and 4-HI (Fig. 2). These results suggested that increases in biofilm formation by indole and 4-HI were associated with eDNA release. Moreover, a large amount of eDNA may contribute to a dense biofilm architecture under 4-HI added conditions. eDNA plays a role in biological functions other than biofilm formation. The presence of eDNA in biofilms confers increased resistance against aminoglycoside (Wilton *et al.*, 2016). Oral bacteria may utilize eDNA as a nutrient source to survive nutrient-limited conditions and chelating metal ions (Jakubovics and Grant Burgess, 2015). Thus, increases in eDNA following the addition of indoles may be advantageous for the growth of *S. mutans*. Further investigations are needed to elucidate the impact of eDNA release induced by indoles on the ecology of *S. mutans*.

In *S. mutans*, eDNA is released as a consequence of cell death via the intercellular signaling pathway, generally called quorum sensing. Two quorum sensing signals induce cell death and eDNA release in *S. mutans* in a cell density-dependent manner and consist of competence-stimulating peptide (CSP) (Perry *et al.*, 2009) and *sigX*-inducing peptide (XIP) (Wenderska *et al.*, 2012). The *comC* and *sigX* genes encode CSP and XIP, respectively. Therefore, we quantified the biofilm amounts of Δ*comC* and Δ*sigX*, which are strains with deficient CSP production and sigma factor stimulated by XIP, respectively (Fig. 3). The results obtained showed that biofilm formation was not increased by the addition of indole to Δ*comC* and Δ*sigX*. Moreover, since LytF autolysin, the expression of which is induced via quorum sensing pathways, was shown to cause the autolysis of cells (Dufour and Lévesque, 2013), we also examined Δ*lytF* in the biofilm formation assay. The biofilm amounts of Δ*lytF* were significantly increased by the addition of indole. These results indicate that indole induced biofilm formation via CSP and XIP quorum sensing pathways; however, LytF autolysin was not necessary for the stimulation of biofilm formation by indole. Although the interconnection between CSP and the XIP signaling pathway currently remains unclear, the activation of the XIP signaling pathway requires CSP in complex medium (Reck *et al.*, 2015). Therefore, the present results suggest that indole acts through the XIP quorum sensing pathway, but is mediated by an unknown autolysis pathway. Moreover, the addition of 4-HI significantly decreased biofilm formation by quorum sensing mutants, suggesting that 4-HI exhibited growth inhibitory activity independent of quorum sensing pathways. In the wild type, 4-HI inhibited cell growth and decreased maximum cell density at the stationary phase more than DMSO and indole (Supplementary Fig. S2). This inhibitory effect was not restored in the quorum sensing mutants; furthermore, the growth rates of these mutants were suppressed more than the wild type (Supplementary Fig. S2). These results suggest that 4-HI exhibits antibiotic-like activity, resulting in eDNA release independent of quorum sensing. A subminimal inhibitory concentration of antibiotics has been shown to induce eDNA release and eDNA-dependent biofilm formation by Staphylococci (Kaplan *et al.*, 2011; 2012). Similarly, the antibiotic-like activity of 4-HI may induce eDNA release, resulting in the induction of biofilm formation in *S. mutans*. Δ*comC* is more sensitive to some antimicrobial agents, such as ampicillin and chlorhexidine (Wang *et al.*, 2013), suggesting that the quorum sensing cascade confers tolerance to 4-HI. Thus, the growth inhibitory effects of 4-HI may significantly decrease biofilm volumes in quorum sensing mutants.

Although the different effects of indole and its derivatives imply the complexity of interspecies interactions in the oral microbiome, the present study revealed for the first time a potential interaction between cariogenic *S. mutans* and indole-producing bacteria during biofilm formation. Indole is converted to oxidized indole derivatives, such as isatin, 2-HI, 3-HI, and 4-HI, by the enzymatic activity of bacteria (Rui *et al.*, 2005). Our results demonstrated that indole modifications and metabolism were crucially important for the biofilm-stimulating activity of indole, and further studies on the metabolic dynamics of indole in oral microbiota are awaited. Interspecies interactions via signaling molecules may shape mixed oral biofilm formation and the composition of the community, and indole produced by the oral microbiota may be a candidate signaling molecule to control the oral microbiota. To elucidate interspecies interactions among *S. mutans* and oral commensal bacteria, we need to investigate the molecular mechanisms underlying the responses to indole and its derivatives in *S. mutans* during biofilm formation. Furthermore, comprehensive metagenomic, metatranscriptomic, and metabolome analyses of the oral microbiome producing or responding to indole will contribute to a more detailed understanding of oral microbial ecology based on intercellular communications. The saliva of patients with periodontal disease contains high amounts of indole and some periodontopathogenic bacteria, such as *Porphyromonas gingivalis*, *Prevotella* sp., and *F. nucleatum*, which produce indole (Berg *et al.*, 1946; Sasaki-Imamura *et al.*, 2010; Sasaki-Imamura *et al.*, 2011). Further investigations on intercellular interactions in the oral cavity via indole will also provide critical insights into the onset and treatment of oral infectious diseases, dental caries, and periodontitis.

## Citation

Inaba, T., Obana, N., Habe, H., and Nomura, N. (2020) Biofilm Formation by *Streptococcus mutans* is Enhanced by Indole via the Quorum Sensing Pathway. *Microbes Environ ***35**: ME19164.

https://doi.org/10.1264/jsme2.ME19164

## Supplementary Material

Supplementary Material

## Figures and Tables

**Fig. 1.**
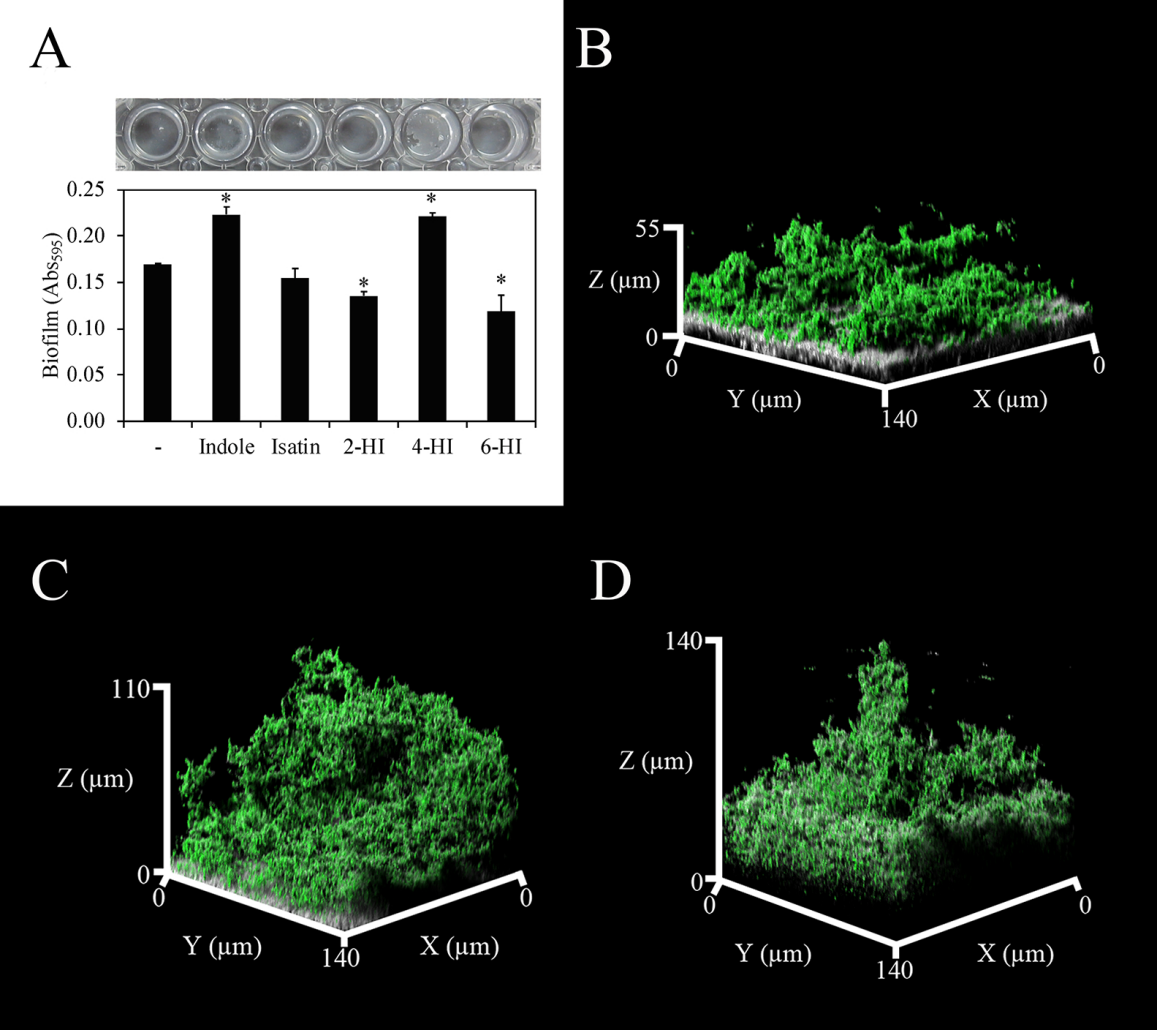
Effects of indole and its derivatives on biofilm formation by *Streptococcus mutans*. Effects of indole and its derivatives on biofilm formation by *Streptococcus mutans* strain UA159 (A). Values represent the means and standard deviations of three biological replicates. Asterisks indicate a significant difference from the DMSO control (shown as “–”) (*P*<0.05), as evaluated by Dunnett’s test (A). A representative of at least three independent experiments is shown. Confocal laser scanning microscopic images of the biofilm grown with DMSO as a control (B), 500‍ ‍μM indole (C), and 4-HI (D). Regarding microscopic visualization, biofilms were formed on the surfaces of hydroxyapatite disks. Green indicates the cells of *S. mutans* labeled by SYTO9, and gray the reflection signals representing non-cell parts, particularly arrows. The gray color at the bottom of the images indicates the surfaces of the hydroxyapatite disks. Visualization of biofilms formed on a non-transparent substratum was performed following a previously described procedure (Inaba *et al.*, 2013), and a detailed procedure is shown in Supplementary materials. Each projection shows fields of 140×140‍ ‍μM (x-y) as indicated. Microscopic images were taken of at least three random positions, and representative images are shown.

**Fig. 2.**
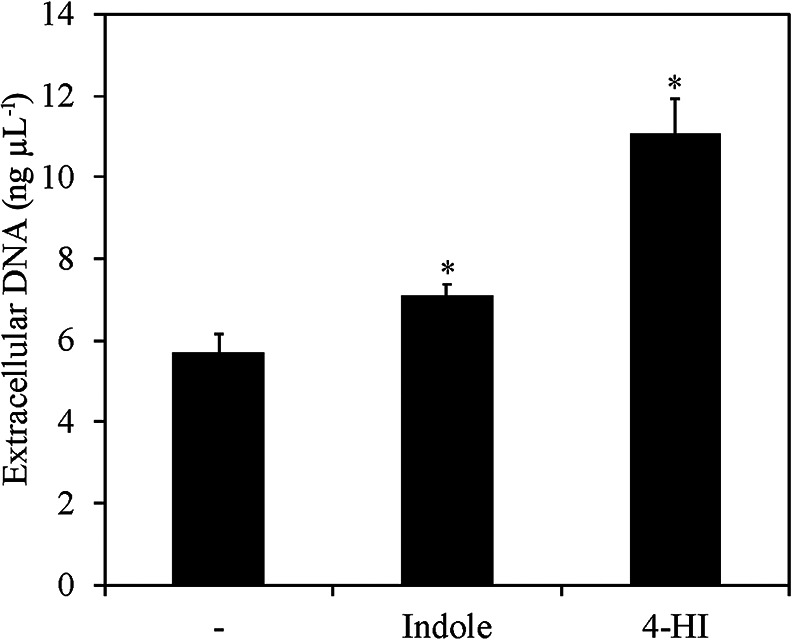
Quantification of eDNA. The amounts of eDNA in biofilms. Cells were grown in TSB medium with or without 500‍ ‍μM indole and 4-HI at 37°C for 12 h in a 24-well microtiter plate. A detailed procedure for eDNA quantification is shown in Supplementary materials. Representative data from two independent experiments are shown in the figure. Values represent the means and standard deviations of at least three biological replicates. Asterisks indicate a significant difference (*P*<0.05), as evaluated by Dunnett’s test.

**Fig. 3.**
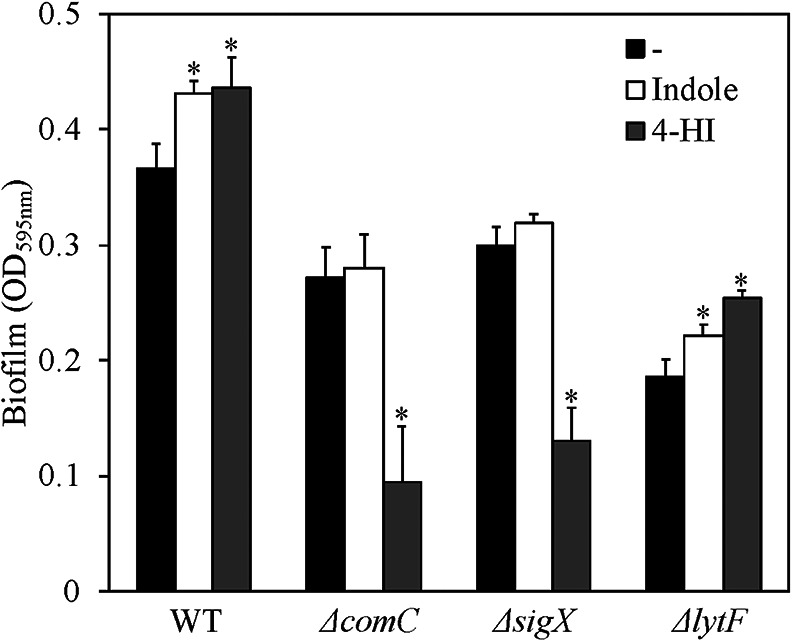
Biofilm assay for quorum sensing mutants. Biofilm amounts of quorum sensing mutants. Biofilms were formed at the bottom of a 24-well microtiter plate. A biofilm assay was performed according with the procedure shown in Supplementary materials. Asterisks indicate a significant difference based on three biological replicates from the DMSO control (*P*<0.05), as evaluated by Dunnett’s test. At least three independent experiments were performed, and representative experiments are shown.
