# Supplementary material for: Biofilm Formation by Streptococcus mutans is Enhanced by Indole via the Quorum Sensing Pathway
Source: Microbes Environ. 2020 Apr 29;35(2):ME19164. doi: 10.1264/jsme2.ME19164 (PMC7308578; doi:10.1264/jsme2.ME19164)
Supplement: Supplementary file 1 — Supplementary Material [file 35_19164_s1.pdf]

Supplementary material for

Biofilm Formation by *Streptococcus mutans* is Enhanced by Indole via the Quorum Sensing Pathway

By Tomohiro Inaba, Nozomu Obana, Hiroshi Habe and Nobuhiko Nomura

Supplementary: Materials and Methods

Bacterial strains and culture conditions. The bacterial strains used in this study are listed in Supplementary Table S1. For routine culture, bacterial strains were grown in Tryptic soy broth (TSB; Becton Dickinson, Sparks, USA) or on TSB agar plates at 37°C aerobically or anaerobically, respectively. For anaerobic incubation, Anaeropack (Mitsubishi Gas Chemical, Tokyo, Japan) was used according to the manufacture’s instruction.

Supplementary Table S1. Strains used in this study

Strains	Relevant properties	Disrupted gene product	Source
NCIMB702062	Wild type	-	JCM
UA159	Wild type	-	Lab stock
FSC3	Wild type, a clinical isolate	-	(1)
Δ*comC*	Erm_R, UA159 derived, *comC* deficient	CSP production	(2)
Δ*sigX*	Erm_R, UA159 derived, *sigX* deficient	RNA polymerase sigma factor	This study
Δ*lytF*	Erm_R, UA159 derived, *lytF* deficient	Autolysin	This study

1. Motegi, M., Y. Takagi, H. Yonezawa, N. Hanada, J. Terajima, H. Watanabe, H. Senpuku. 2006. Assessment of Genes Associated with *Streptococcus mutans* Biofilm Morphology. Applied and Environmental Microbiology 72:6277.
2. Tamura, S., H. Yonezawa, M. Motegi, R. Nakao, S. Yoneda, H. Watanabe, T. Yamazaki, H. Senpuku. 2009. Inhibiting effects of Streptococcus salivarius on competence-stimulating peptide-dependent biofilm formation by *Streptococcus mutans*. Oral Microbiology and Immunology 24:152-161.

Construction of the mutant strains. We constructed the mutant strains as follows. The DNA fragments of the 5' and 3' flanking regions of the target gene were ligated to the erythromycin resistance gene by overlap extension PCR. The resultant PCR products were introduced into S. mutans cells by the natural competence method. The transformants were selected on brain heart infusion (BHI) agar containing 10 g/ml of erythromycin. Each mutant was confirmed through PCR and DNA sequencing.

Biofilm formation assay and microscopic observation. For the biofilm formation assay, cells were grown in TSB medium at 37°C for 12 hours with or without 0.5, 5, 50 and 500 μM indole (Fujifilm Wako Pure Chemical Industries, Ltd., Osaka, Japan) and its derivatives (Fujifilm Wako). Indole and derivatives were dissolved in dimethyl sulfoxide (DMSO), and DMSO served as a control. Biofilms on the bottom of a 24-well microtiter plate were washed twice with distilled water and then resuspended in water for the quantification of turbidity as an amount of biofilm by spectrophotometer. For microscopic visualization, biofilms were formed on the surface of hydroxyapatite disks (Clarkson

Chromatography Products Inc., PA, US). Twelve-hour-old biofilms were washed once with water and then stained with 5 μM SYTO9 (fin. conc., Molecular Probes, OR, US) for bacterial cell labeling. The stained biofilm sample was washed with distilled water once, and then, was put at the bottom of the chamber filled with distilled water. A laser scanning microscope (LSM5 and 880; Carl Zeiss, Jena, Germany) equipped with a 63x numerical aperture Achroplan W water immersion objective (Carl Zeiss) was used to acquire the biofilm images.

Quantification of extracellular DNA. Cells were grown in TSB medium with or without 500 μM indole and 4-HI at 37°C for 12 hours in a 24-well microtiter plate. Culture supernatants were removed, and biofilms on the bottom of each well were suspended in 1 ml of distilled water. Biofilm suspensions were separated again by centrifugation, and then eDNAs were purified from the supernatants by phenol/chloroform extraction and ethanol precipitation. The concentrations of extracted eDNA were quantified by absorbance at 260 nm.

Supplementary Figures

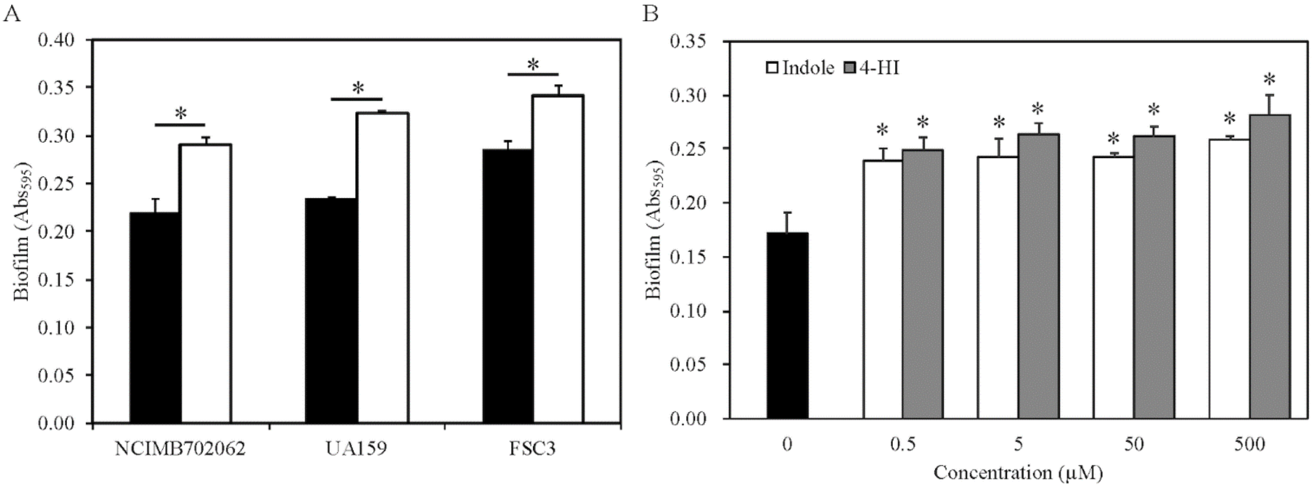

Supplementary Figure S1. Effect of indole on three *S. mutans strains* and impact of concentrations on biofilm formation of UA159. (A) The effect of indole on the biofilm formation of *S. mutans* strains. (B) Influence of indole and its derivative concentrations on biofilm formation. The values represent the means and standard deviations of three biological replicates. Asterisks indicate statistical significance relative to the DMSO control (*P*-value <0.05), as evaluated by unpaired two-tailed student's *t*-test (A) and Dunnett's test (B). A representative of at least three independent experiments is shown.

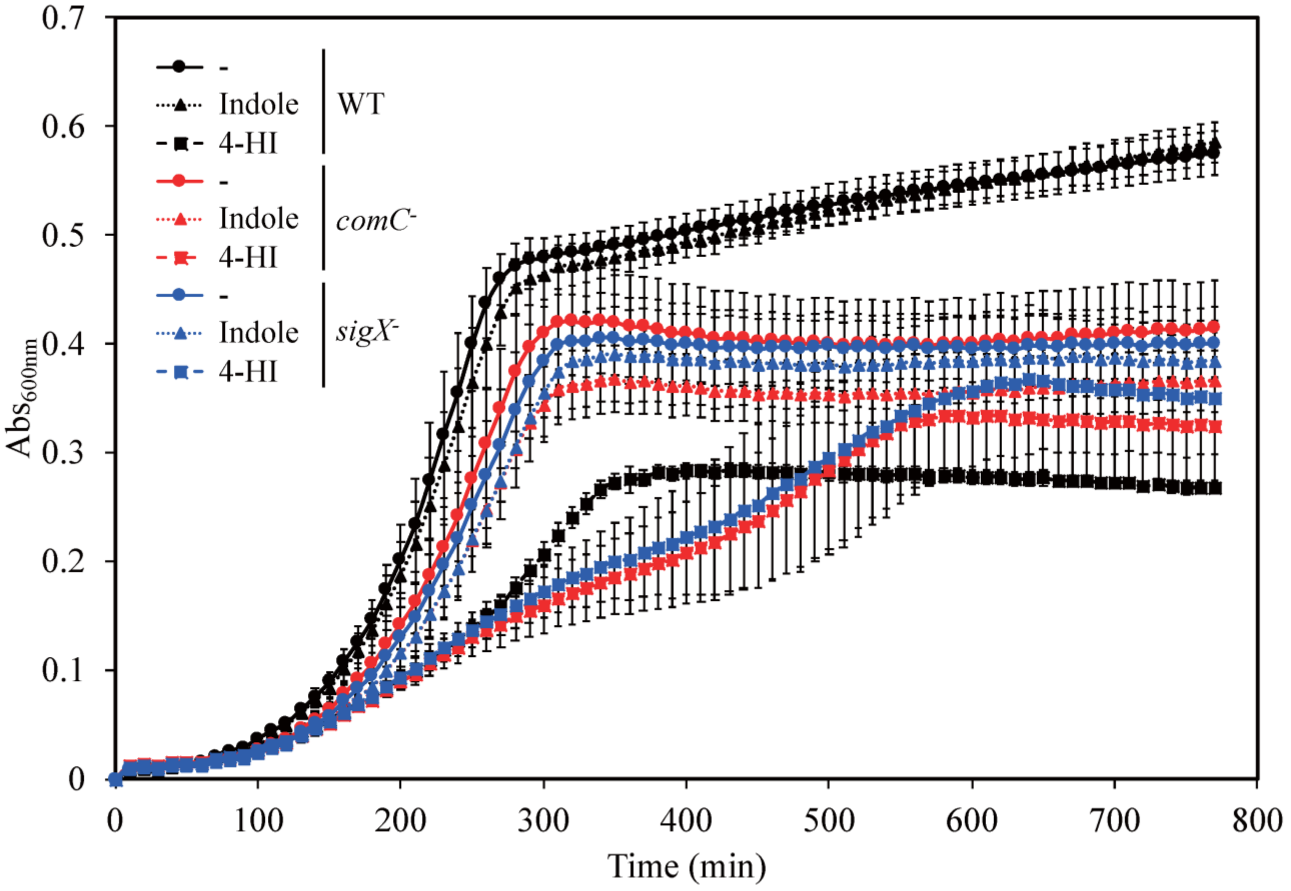

Supplementary Figure S2. Growth curve of *S. mutans* cultured with or without indole and 4-HI. Wild type, *comC* and *sigX* mutants were statically cultured in 96-well plate filled with TSB medium containing 500 μM indole or 4-HI at 37°C. Indole and 4－HI were dissolved in DMSO, which served as a control (shown as "-"). The absorbance at 600 nm was measured every 10 minutes for 12 hours by a microplate reader (Varioskan Flash, Thermo Scientific, Massachusetts, US). The values indicate the average of eight biological replicates, and bars indicate standard deviations. At least three independent experiments were performed, and representative data are shown in the figure.
